# Seven Years of Selective Genetic Screening Program and Follow-Up of Asymptomatic Carriers With Hereditary Transthyretin Amyloidosis in Bulgaria

**DOI:** 10.3389/fneur.2022.844595

**Published:** 2022-04-08

**Authors:** Teodora Chamova, Mariana Gospodinova, Ognian Asenov, Tihomir Todorov, Zornitsa Pavlova, Andrey Kirov, Sylvia Cherninkova, Kristina Kastreva, Ani Taneva, Stanislava Blagoeva, Sashka Zhelyazkova, Plamen Antimov, Kaloian Chobanov, Albena Todorova, Ivailo Tournev

**Affiliations:** ^1^Department of Neurology, Expert Centre for Hereditary Neurologic and Metabolic Disorders, University Hospital “Alexandrovska”, Medical University-Sofia, Sofia, Bulgaria; ^2^Expert Center for Transthyretin Cardiac Amyloidosis, University Hospital “St Ivan Rilski”, Sofia, Bulgaria; ^3^Independent Medico-Diagnostic Laboratory Genome Center “Bulgaria”, Sofia, Bulgaria; ^4^Genetic Medico-Diagnostic Laboratory Genica, Sofia, Bulgaria; ^5^Department of Medical Chemistry and Biochemistry, Medical University Sofia, Sofia, Bulgaria; ^6^Department of Cognitive Science and Psychology, New Bulgarian University, Sofia, Bulgaria

**Keywords:** ATTRv, screening, asymptomatic carrier, follow up, phenotype

## Abstract

Hereditary transthyretin amyloidosis (ATTRv amyloidosis) is a rare, autosomal-dominant (AD) multisystem disorder resulting from the extracellular deposition of amyloid fibrils formed by a destabilized mutant form of transthyretin (TTR), a transport protein predominantly produced by the liver.

**Aim:**

The aims of the current study are to demonstrate the Bulgarian experience with the screening programs among the high-risk patient population over the last 7 years, to present the results from the therapy with TTR stabilizer in our cohort, as well as to stress on the importance of a follow-up of asymptomatic carriers with TTR pathogenic variants by a multidisciplinary team of specialists.

**Materials and Methods:**

In 2014, a screening program among the high-risk patient population for ATTRv was initiated in Bulgaria. On one hand, it was conducted to identify new patients and families among people with “red flag” clinical features, while on the other hand, the program aimed to identify TTR mutation carriers among the families with already genetically proven diagnoses. Sanger sequencing methodology was used to make fast target testing for mutations in the *TTR* gene in the suspected individuals. All of the identified carriers underwent subsequent evaluation for neurological, cardiac, gastroenterological, and neuro-ophthalmological involvement. Those considered affected were provided with multidisciplinary treatment and a follow-up.

**Results:**

As a result of a 7-year selective screening program among the high-risk patient population and relatives of genetically verified affected individuals, 340 carriers of TTR mutations were identified in Bulgaria with the following gene defects: 78.53% with Glu89Gln, 10.29% with Val30Met, 8.24% with Ser77Phe, 2.06% with Gly47Glu, and 0.59% with Ser52Pro. All of these affected displayed a mixed phenotype with variable ages at onset and rate of progression, according to their mutation. From the 150 patients treated with TTR stabilizer, 84 remained stable, while in other 66 patients the treatment was terminated either because of polyneuropathy progression or due to death. A program for a regular follow-up of asymptomatic carriers in the last 3 years enabled us to detect the transition of 39/65 to symptomatic patients and to initiate treatment in a timely manner.

**Conclusion:**

Bulgarian ATTRv patients display a mixed phenotype with some clinical peculiarities for each mutation that should be considered when treating the affected and the follow-up of the asymptomatic carriers of a specific gene defect.

## Introduction

Hereditary transthyretin amyloidosis (ATTRv amyloidosis) is a rare and debilitating multisystem disorder resulting from the extracellular deposition of amyloid fibrils formed by a destabilized mutant form of transthyretin (TTR), a transport protein predominantly produced by the liver (1, 2). It has an autosomal-dominant (AD) trait of inheritance and is associated with more than 140 different mutations in the *TTR* gene (1–5). The age at onset is highly variable from the second to the ninth decade of life (6), and the clinical phenotype varies from a progressive sensorimotor and autonomic neuropathy to an infiltrative cardiomyopathy (1, 6).

Current disease-modifying therapies for ATTRv that aim to stabilize the TTR tetramer (Diflunisal and Tafamidis) or to reduce mutant TTR production (liver transplantation or gene-silencing therapies) (7) are associated with improved outcomes when initiated early in the disease course, therefore timely diagnosis is crucial.

Bulgaria is among the countries with a high number of ATTRv amyloidosis cases. The most common TTR pathogenic variant for the Bulgarian patients is Glu89Gln (NM_000371.3: c.325G>C, p.Glu109Gln, rs121918082) (8–10), but four other less prevalent mutations have been found as well—Val30Met (NM_000371.3: c.148G>A, p.Val50Met, rs28933979), Ser77Phe (NM_000371.3:c.290C>T, p.Ser97Phe), Gly47Glu (NM_000371.3:c.200G>A, p.Gly67Glu, rs121918090), and Ser52Pro (NM_000371.3: c.214T>C, p.Ser72Pro). The founder effect has been proven for some of the TTR mutations in Bulgaria (10, 11).

The expert ATTR Amyloidosis Centre at the University Hospital “Alexandrovska” in Sofia, Bulgaria, was set up in early 2016 and it is currently a member of the European Reference Network for Neuromuscular Disease (ERN-EURO NMD) (12). The Bulgarian multidisciplinary team in this center of excellence (CoE) encompasses different specialists, dealing with the diverse aspects of this disorder: neurologists, ophthalmologists, cardiologists, gastroenterologists, nephrologists, geneticists, physiotherapists, psychologists, pathologists, and nurses. The CoE has been performing a screening program for diagnosing ATTRv in the high-risk patient population over the last 7 years (9) and a program for a follow-up of asymptomatic carriers of mutations in the *TTR* gene since 2018.

The aims of the current study are to demonstrate the Bulgarian experience with the screening programs among the high-risk patient population over the last 7 years, to present the results from the therapy with TTR stabilizer in the Bulgarian cohort, as well as to stress on the importance of a follow-up of asymptomatic carriers with *TTR* pathogenic variants by a multidisciplinary team of specialists.

## Materials and Methods

### Selective Screening Program

Early diagnosis is critical for the effectiveness of the treatment and the quality of life of patients. In this respect, in 2014 a screening program among the high-risk patient population for ATTRv was initiated in Bulgaria. On one hand, it was conducted to identify new patients and families among people with the following clinical features: bilateral carpal tunnel syndrome and/or axonal polyneuropathy of unknown cause and/or restrictive cardiomyopathy with infiltrative features and/or diarrhea/constipation/weight loss. The family history, consistent with AD trait of inheritance and origin from the already identified endemic regions were added as inclusion criteria as well. On the other hand, the program aimed to identify *TTR* mutation carriers among the families with already genetically proven diagnoses.

A public awareness campaign was organized. Our CoE team made the medical specialists familiar with the aims and stages of the selective screening program by organizing regional and national meetings with the participation of general practitioners (GPs), different specialists (neurologists, cardiologists, and gastroenterologists). Nine common meetings with the ATTRv patient organization were performed during this 7-year period. All participants in the screening were informed in writing about their carrier status, and further consultations were provided individually. Genetic counseling was offered to all of the detected carriers.

The members of the team were performing field studies and were visiting the affected families in their towns and villages. This “door to door” information campaign had the following main standpoints: what the disease itself is, how it could appear, how it could be inherited and transmitted to the offspring, what the genetic analysis itself is, what could the detected carriers expect, how often must the carriers be followed according to the age, and when the treatment should be initiated. A patient manual, describing all of the above issues was elaborated and provided to those affected and their families.

The genetic diagnostic approach in Bulgaria started with the evaluation of the frequency and the geographic distribution of the most common *TTR* mutations. We used the Sanger sequencing methodology, which allowed us to make fast target testing of the suspected individuals. Based on this, the genetic screening started with sequencing of exon 3 of the *TTR* gene as the most frequent mutations Glu89Gln and Ser77Phe are located there. If exon 3 was negative for pathogenic variants, exon 2 was sequenced for Val30Met and Gly47Glu. In case of negative target screening, we proceeded with a full study of the coding sequence of the *TTR* gene. As long as all the known amyloidogenic mutations are located in the *TTR* coding sequence, this test was enough to confirm or exclude the hereditary form of ATTR.

All of the genetically verified patients underwent clinical assessment at the time of the diagnosis and a routine follow-up every 6 months, including neurological examination, The neuropathy impairment score (NIS) (13), evaluation of familial amyloid polyneuropathy (FAP) stage, and polyneuropathy disability score (PND) (14). Neurophysiological assessments encompassed electromyography with nerve conduction studies (NCSs), sympathetic skin response (SSR), and electrochemical skin conductance (ESC), measured by Sudoscan (15). Motor (tibial, peroneal, median, and ulnar) and sensory (sural, superficial peroneal, median, and ulnar) nerve conductions (NCVs) were assessed on both sides of the body. The SSR was recorded with electrodes on the hand (and foot) dorsum and on the corresponding sites of palm and sole. Peak-to-peak amplitude was assessed (16).

Cardiac involvement was evaluated in all of the affected patients by 12-lead electrocardiography (ECG) and transthoracic echocardiography. In patients positive for ATTRv, cardiac involvement was defined echocardiographically as an end-diastolic left ventricular (LV) wall thickness more than 12 mm in the absence of any other causes for ventricular hypertrophy according to a consensus (17). The presence of infiltrative features, such as interatrial septal and valve thickening, as well as the presence of apical sparing, characteristic ECG findings supported the diagnosis. 99mTc-PYP bone scintigraphy and, in some of the patients, cardiac magnetic resonance (CMR) were performed when the findings from the echocardiography exam were equivocal. An ECG was considered abnormal when it revealed one or more of the following signs: different from sinus rhythm and conduction disturbances (right bundle branch block, left bundle branch block, A–V block, left anterior fascicular block, left posterior fascicular block, and unspecific intraventricular delay), low voltage of the QRS complex, deviations in the ST segment and *T* wave, pathological *Q* wave, or a prolongation of the QT interval. Low-voltage amplitude in the limb leads was defined by a mean QRS amplitude in leads I, II, III, aVL, and aVF of 0.5 mV or <1 mV in all precordial leads (8). Complete echocardiographic examinations, including 2D, M-mode, spectral and color Doppler techniques, tissue Doppler imaging (TDI), and 2D speckle tracking echocardiography (STE), were done in all patients. The echocardiographic studies were performed and recorded, and all the measurements were done in accordance with internationally approved standards (18–20).

Gastrointestinal involvement was assessed in all by taking patients' history, biomarkers for intestinal inflammation (fecal calprotectin), hydrogen breath test, abdominal ultrasound, and endoscopies with staging biopsies for Congo red staining (12, 21).

In total, 69 patients with ATTRv (40 men and 29 women) were ophthalmologically examined in the Clinic of Nervous diseases, Neuro-ophthalmology office, Sofia, by one senior ophthalmologist. The ophthalmological examination consists of the test for visual acuity, slit-lamp examination of the anterior eye segment, pupil function, slit lamp examination of the ocular fundus using +90D lens, an examination of lacrimal function using Schirmer's test and tear break-up time (TBUT), and intraocular pressure and ocular motility was performed.

### Asymptomatic Carriers

During our COE's genetic screening program, the asymptomatic adult family members of already verified ATTRv cases had an opportunity for further evaluations, which allowed us to perform structured monitoring of individuals who carry a mutant *TTR* gene and enhanced the possibility of early diagnosis of the disease (22). Genetic counseling allowed the family members to make an informed decision about genetic testing for *TTR*.

All these asymptomatic carriers were invited for neurological evaluation, NCS, SSR, Sudoscan, and cardiac evaluation with ECG, echocardiography, and in case of suspicion for cardiac involvement-−99mTc-PYP bone scintigraphy with a frequency, according to their age and mutation. The transition from asymptomatic to the symptomatic patient was based on the already approved European guidelines (22).

## Results

### Screening Program

The first ATTRv family was diagnosed in Bulgaria in 2008.

**Figure 1:**
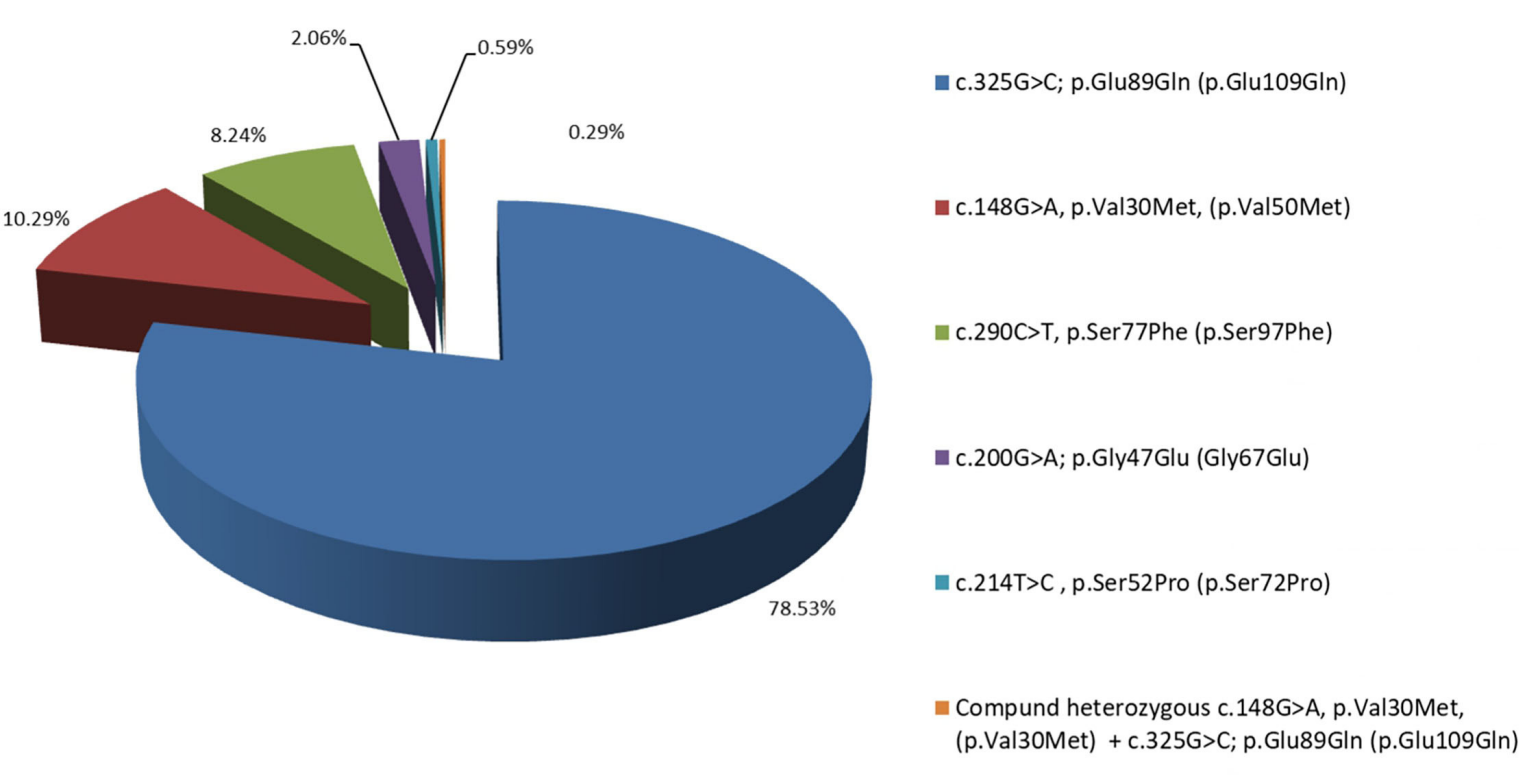
Distribution in percentage of the Bulgarian hereditary transthyretin amyloidosis (ATTRv) patients, according to their mutation: 78.53% with Glu89Gln, 10.29% with Val30Met, 8.24% with Ser77Phe, 2.06% with Gly47Glu, and 0.59% with Ser52Pro.

In the 7 years of the screening program (from 2014 to 2021), 954 people fulfilling the above-mentioned inclusion criteria were tested and 340 carriers (179 male and 161 female) of TTR mutations both affected and asymptomatic belonging to 127 pedigrees were identified Figure 1. They are distributed according to the TTR mutation as follows: 267 (78.53%; 140 male and 127 female) with Glu89Gln; 35 (10.29%; 22 male and 13 female) with Val30Met; 28 (8.24%; 13 male and 15 female) with Ser77Phe; 7 (2.06%; 4 male and 3 female) with Gly47Glu; 2 (0.59%; 2 female) with Ser52Pro, and 1 with a compound heterozygous carrier of Val30Met and Glu89Gln. In total, 470 of the tested individuals belonging to these 127 pedigrees did not carry any mutation in the TTR gene.

The calculated average carrier frequency of the *TTR* mutations in the screened Bulgarian families corresponds with the AD pattern of inheritance with a theoretical risk of transmission of 50%. Despite the particular mutations, the frequency of the TTR mutation carriers from all screened individuals originating from ATTRv-affected families is 42% (Supplementary Table 1).

Of the remaining 144 patients, who neither belonged to an ATTRv family, nor were carrying a *TTR* mutation 134 had a late-onset axonal polyneuropathy, while the other 10 displayed restrictive cardiomyopathy with infiltrative features.

Epidemiological data of the affected patients are presented in Table 1, while the predominant clinical features according to patients' mutation are presented in Table 2.

**Table 1:** Epidemiological data of Bulgarian hereditary transthyretin amyloidosis (ATTRv) patients.

**Mutation in *TTR* gene**	**Number of patients**	**Mean age at onset (years)**	**Range of age at onset (years)**	**Median survival (years)**	**Mean time from symptoms onset to diagnosis (years)**
**Glu89Gln**					
Total	184	51.21	39–69	11.55 ± 0.83	3.67 ± 3.26
Male	90	48.33	39–58	10.65	3.04 ± 2.03
Female	94	54.09	43–69	11.87	4.34 ± 4.10
**Val30Met**					
Total	27	66.66	54–74	8.98	3.65 ± 2.84
Male	18	64.94	54–73	8.98	4.03 ± 2.90
Female	9	68.38	62–74	No deceased females	2.1 ± 1.9
**Ser77Phe**					
Total	12	58.34	50–64	8.30 ± 0.82	2.75 ± 1.71
Male	6	56.76	50–62	7.30 ± 0.82	2.8 ± 1.93
Female	6	59.92	53–64	9.00	2.67 ± 1.24
				Only one deceased female	
**Gly47Glu**					
	6	29.15	18–38	4.68 ± 0.41	2.67 ± 1.70
Male	3	28.5	18–32	4.58	3.55 ± 1.12
Female	3	29.8	18–38	No deceased females	2.01 ± 1.18

**Table 2:** Semiquantitative overview of clinical presentation frequency.

**Mutation in *TTR* gene**	**Neurological presentation**			**AD**	**CI**	**GI**	**Weight loss**
	**PNP**	**SFN**	**CTS**				
Glu89Gln	+++	+++	+++	+	+++	++	++
Val30Met	+++	++	++	++	+	+	++
Ser77Phe	++	++	++	+	++	++	++
Gly47Glu	+	+++	+	+++	++	+++	+++

*(+ is uncommon or mild, + + is average or moderate; + + + is frequent or intense)*.

*SFN, small fiber neuropathy; CTS, carpal tunnel syndrome; AD, autonomic dysfunction; CI, cardiac involvement; GI, gastrointestinal involvement*.

*Glu89Gln* was found to be the most frequent *TTR* mutation in Bulgaria (78.53%), with an endemic region, consisting of two districts: Blagoevgrad and Kjustendil, in the south-western part of the country. The focus is 200 km long and is located along the border with the Republic of Northern Macedonia. Due to internal migrations, patients with this mutation were found in the north-eastern part of Bulgaria as well. They were less frequently identified in central Bulgaria. The region is referred as endemic based on the data from National Statistical Institute because the incidence is estimated to be 1/7,636, while the prevalence is 1/4,150 (23).

There is a statistically significant difference in the mean age at onset between male symptomatic carriers with Glu89Gln (48.33 years varying between 39 and 58 years) and female patients (54.09 years, varying between 43 and 69 years). The median survival was 11.55 ± 0.83 years, ranging between 3 and 20 years (23). The clinical phenotype encompassed: bilateral carpal tunnel syndrome; sensory, motor, and autonomic neuropathy with painful dysesthesias more pronounced in the feet; the loss of vibration and temperature sensations; sensory ataxia, autonomic dysfunction; distal muscle weakness; restrictive cardiomyopathy with heart failure signs and symptoms, rhythm and conduction disturbances, and syncope; and constipation/diarrhea, weight loss, and subsequent cachexia.

Polyneuropathy was considered as the predominant initial symptom. Rarely, peripheral nerve, cardiac, and gastrointestinal symptoms were observed simultaneously in the beginning of the disease, thus broadening the differential diagnosis. In only 30 of the patients, cardiac symptoms were reported prior to other symptoms for years. Patients with Glu89Gln developed earlier and more severe restrictive cardiomyopathy compared to patients with Val30Met. Gastrointestinal involvement was found as an initial sign-in for only 7 of the affected patients.

Decreased tear secretion (<10 mm moisture of the paper strip for 5 min) was found in 14/23 patients (28 eyes, 60.9%). Abnormal TBUT test (an initial break-up of the tear film <6 s) was found in 8/14 patients (16 eyes, 44.4%). In the slit lamp examination, suspected bilateral amyloid deposition on the anterior capsule of the lens was found in only one patient with ATTRv—a 68-year-old female (2 eyes, 1.4%) with Glu89Gln mutation of the *TTR* gene. Vitreous amyloidosis was found in two examined ones. The vitreous opacities were with the characteristic of fibrils, rounded and veil-like condensates, or by the type of pseudopodia lentis. No severe functional opacity-related visual deficits were observed in any of the affected patients and did not require vitrectomy to confirm the amyloid immunohistologically. In two female patients with ATTRv, about 7 years after disease onset, bilateral ptosis, and mild unilateral abducens nerve lesion were observed. Brain magnetic resonance imaging (MRI) was normal in both.

The clinical phenotype of our *Glu89Gln* group was mixed, with the involvement of the peripheral nerves, heart, and gastrointestinal system.

In total, 14 affected families with *Val30Met* were identified in Bulgaria. In general, the distribution of patients with this mutation across the country is more widespread. However, there is also a small endemic region in the Smolyan district.

The incidence of the mutation in the endemic region is 1/9,000 and its prevalence is 1/3,200 (23). Reports of positive family history were present in <25–30% of the affected patients, thus often the disease was interpreted as “sporadic.”

The clinical phenotype was of a late-onset disease with a mean age at the onset of 65.57 years (age range: 55.4–76 years). The median survival was 8.98 years (range: 6–9 years). All of the patients had axonal sensory-motor and autonomic polyneuropathy associated with LV hypertrophy and left atrial enlargement. Most of them had alternating constipation-diarrhea, impotence, orthostatic hypotension, and urinary incontinence as a later clinical feature. Vitreous amyloidosis was found in only one patient.

At diagnosis clinical phenotype was mixed—peripheral nerve and cardiac involvement with nerves predominantly affected. Clinically, the phenotype was “milder,” compared to the other mutations described in Bulgaria.

In total, 12 affected families with *Ser77Phe* were identified in Bulgaria, all of them originating from the village of Vakarel, located 25 km from the capital of Bulgaria, Sofia. Positive family history was reported in 85% of the cases. The incidence of the mutation in the endemic region is 1/1,600 and its prevalence is 1/850 (23).

Clinical phenotype is of a late-onset form with a mean age at onset 57.60 years. (52.3–61.8 years). Median survival was 7.30 ± 0.82 years (ranging from 4 to 9 years). All the patients had axonal sensory-motor and autonomic polyneuropathy associated with restrictive cardiomyopathy. The majority of them had alternating constipation-diarrhea, impotence, orthostatic hypotension, and urinary incontinence as late clinical features. In only one affected vitreous amyloidosis was observed.

The clinical phenotype at diagnosis was mixed, most often neuropathic and cardiac, while gastrointestinal involvement was observed in the advanced stages. The phenotype was considered as similar to Glu89Gln.

Two large Roma families, carrying the mutation *Gly47Glu* in three generations were identified in the Russe district in the north-eastern part of the country. The average age at onset of the disease was 28.5 years (18–38 years) in the last generation. There were data for four men who have died between the ages of 30 and 35 years. In the first and the second generations, the disease has started later (average age at the onset−40 years) and three patients have died at 46 years of age. Mean survival was estimated as 4.68 ± 0.41 years, ranging from 4 to 6 years. Genetic anticipation in the age at onset of the disease was observed. The presenting symptoms were pain in the feet, nausea, vomiting, weight loss up to 50 kg, and diarrhea. The clinical course was rapidly progressive.

The clinical phenotype encompassed early and severe gastrointestinal manifestations (severe vomiting and alternating constipation-diarrhea), weight loss, distal and prominent weakness and hypotrophy of all limbs, tendon arreflexia in the lower limbs, decreased vibration sensation in lower limbs, early impotence, severe orthostatic hypotension, restrictive cardiomyopathy, heart failure, and microalbuminuria. The clinical phenotype at diagnosis was mixed, with gastrointestinal, cardiac involvement, and polyneuropathy. Kidney involvement was found as well in three affected.

One affected patient with *Ser52Pro* from Veliko Tarnovo region was identified. The patient had a mother with the same disease, who had died at 63 years. His age at onset was 44.2 years. He died at 53 years. The clinical phenotype at diagnosis was mixed, most often neuropathic and cardiac with GI involvement in more advanced stages. It included axonal sensory-motor and autonomic polyneuropathy, diarrhea, weight loss, severe orthostatic hypotension, restrictive cardiomyopathy, heart failure, albuminuria, renal insufficiency, and secondary anemia.

In the country with a population of 7.3 million, the average incidence of new cases for all mutations and clinically diagnosed patients for a 7-year follow-up is estimated to be 1/42,000. The prevalence of the disease averages 1/26,000.

The most common misdiagnosis in our group with initial neurological involvement were bilateral carpal tunnel syndrome, chronic inflammatory demyelinating polyneuropathy (CIDP), diabetic polyneuropathy, toxic polyneuropathy, cervical, and lumbosacral radiculopathy. In total, 48 affected had a medical history of prior diagnostic errors. They were distributed as follows: idiopathic carpal tunnel syndrome (18%); cervical radiculopathy (12%), lumbosacral radiculopathy (16%), chronic inflammatory demyelinating polyradiculoneuropathy (14%), idiopathic axonal polyneuropathy (10%), toxic (alcoholic) polyneuropathy (10%), diabetic polyneuropathy (4%), vitamin B12-deficient polyneuropathy (4%), drug-induced polyneuropathy (2%), and polyneuropathy associated with gluten sensitivity (2%).

The patients with predominant cardiac phenotype had received the following misdiagnosis—hypertensive heart disease when there was mild to moderate LV hypertrophy or hypertrophic cardiomyopathy with more severe hypertrophy. In total, 11 patients (22.9%) with predominant cardiac phenotype and severe LV hypertrophy (>15 mm) had been misdiagnosed with hypertrophic cardiomyopathy, in 2 of them intraventricular gradient was found, 1 patient had been tested also for Fabry disease. One patient with severe LV hypertrophy had undergone aortic valve replacement and the extra-cardiac symptoms were the clues for ATTR amyloidosis. Mild to moderate hypertrophy had been associated with concomitant arterial hypertension.

In patients with initial gastrointestinal symptoms, the misdiagnoses were distributed as follows: chronic colitis (29%), irritable bowel syndrome (11%), chronic atrophic gastritis (4%), reflux esophagitis (4%), polyp of the sigmoid colon (4%), rectal carcinoma (4%), and pyloric stenosis (2%).

In the first 2 years, around 67% of the diagnosed were in FAP stage 2 or 3 with a diagnostic delay 5–6 years, which was related to poor outcome. Currently, the increased awareness among medical specialists and the identification of different endemic regions for each mutation across the country decreased the diagnostic delay to <2 years after the initial symptoms.

### Follow-Up of Asymptomatic Carriers

For the last 3 years, 65 carriers of TTR mutations were followed up in accordance with the approved recommendations for age and frequency (22). According to their mutations, they were subdivided into the following groups: 56 carriers of Glu89Gln, 3 carriers of Val30Met, 4 carriers of Ser77Phe, and 2 carriers of Gly47Glu.

During this period, 39 of them transitioned to symptomatic with FAP stage I and the PND score 1. In 38 people, abnormalities from the neurological examination, consistent with either bilateral carpal tunnel syndrome or sensory-motor polyneuropathy with attenuated Achillis tendon reflexes and distal hypesthesia in the lower limbs were reported. In 33 of these carriers, the NCS were abnormal with sensory nerve action potential- abbreviation (SNAP) of the sural and the superficial peroneal nerves being decreased or absent in 30 and distal latencies of the median nerve being prolonged for the SNAP and/or compound motor action potential (CMAP). Sudoscan revealed a mild-to-moderate decrease of the electrochemical conductance in 31/38, while SSR was abnormal in all of them. Cardiac impairment was observed in 19/39.

For the period 2014–2021 in 150 patients at FAP stage I, the treatment with Tafamidis 20 mg was initiated. During this period, 84 affected patients remained stable in terms of neurological and cardiologic involvement and continued their treatment with the TTR stabilizer.

The treatment with Tafamidis was terminated in other 66 patients either because of polyneuropathy progression or due to death. Those affected were distributed as follows: 2 patients with Ser77Phe (1.3% from all the treated patients), 3 with Val30Met (2%), 2 with Gly47Glu (1.3%), and 59 with Glu89Gln (39.3%). In the 34 affected patients, the progression of the sensory-motor and autonomic polyneuropathy and transition to FAP stage II were observed and were subsequently switched to gene-silencing therapies. Although all the patients were in FAP stage I at the time of treatment initiation, the heart involvement varied broadly. Clinical manifestations were consistent in 11 patients with NYHA class I, in 20 with NYHA class II, and in 30 patients with NYHA class III. In total, 37 patients demonstrated stage III LV diastolic dysfunction on echocardiography and 32 affected have died: 2 with Ser77Phe mutation and 2 with Val30Met have died suddenly, 1 patient with Gly47Glu has died from gastrointestinal bleeding and the remaining 27 patients were with Glu89Gln mutation-−15 patients have died with advanced heart failure, 7 of them died suddenly, 3 from stroke, 4 from disease progression—bedridden with cachexia and severe autonomic dysfunction, 5 patients from extra-cardiac causes: 1 from esophageal cancer, 1 from acute renal failure, 1 from urosepsis, and two from COVID-19 infection.

Rehabilitation as a key element of the multidisciplinary treatment process of ATTRv was provided to all of the affected patients. It was followed up and updated every 6 months, according to the needs of the affected patients in our CoE.

### Psychological Support

As the diagnosis of ATTRv is often delayed, all patients often share their frustration in seeing many doctors without exact diagnosis and treatment. Psychological support is needed and directed to the patients, carriers, and their families. The patients must have available psychological space in which they can share their feelings and thoughts related with the diagnostic of the disease and managing of the disease. These patients bear a significant emotional burden, a sense of doom, the fear of death, and often a sense of guilt. In most of the cases, these experiences are not shared with their relatives, which further aggravate the situation of patients and lead to internal self-isolation. The psychological support provides a safe and relaxed environment in which patients can talk freely about their condition. Furthermore, it helps the acceptance of the disease and improves their self-perception and the aim of relieving the feeling of being sick.

## Discussion

Hereditary transthyretin amyloidosis is the most serious hereditary polyneuropathy of adult-onset and a progressive, devastating, and life-threatening disease. Timely and appropriate diagnosis can be relatively difficult because the initial presenting symptoms may be vague and attributed to other more common conditions (2, 6, 24). The affected with polyneuropathy have been misdiagnosed with CIDP (25), diabetic polyneuropathy, toxic polyneuropathy, etc. Patients with predominant cardiac phenotype had received several misdiagnoses—hypertensive heart disease when there was mild to moderate LV hypertrophy or hypertrophic cardiomyopathy with more severe hypertrophy (8). Delays in the diagnosis of ATTR can have serious consequences for patients who may develop severe end-organ impairment. In this respect, selective screening programs among the high-risk patient population have proven to be a valuable tool for early diagnosis and timely treatment in the field of rare disorders (2).

For the last 7 years, out of the 954 tested, the Bulgarian genetic selective screening program for ATTRv resulted in the identification of 340 carriers of TTR mutations. In the first 2 years, many of the diagnosed were in FAP stage 2 or 3, which was related to a poor outcome. However, subsequently, increased awareness among medical specialists and the identification of different endemic regions for each mutation across the country decreased the delay in diagnosis.

The data obtained from this research indicated that there were endemic regions in the country of with different areas for individual mutations—Glu89Gln, Ser77Phe, and Val30Met. The incidence and prevalence of the three mutations regarding the place of origin of patients fulfilled the criteria for the endemic areas—between 1/1,000 and 1/10,000 (23).

Currently, there is no Bulgarian genomic database. Available genetic data for the Bulgarian population could be found either in the literature data or in some of the international databases. For example, the GnomAD v.2.1.1 (26) database contains data for Bulgarian exomes and genomes. The Val30Met mutation is found there with a frequency of 0.0003745. However, the publicly available genetic data for the Bulgarian population is far from sufficient, so our medical and scientific community hopes that a database with more complete genetic data will be built in the near future. Glu89Gln as the most frequent TTR mutation in Bulgaria (78.53%) was proven to be a specific regional Balkan-Mediterranean mutation, identified in the Republic of North Macedonia, Turkey, Bulgaria, Kosovo, and Italy (Sicily) (5, 11, 23, 27). The age at onset did not differ significantly between the above-mentioned countries (5, 27). In a comparison study from the Transthyretin Amyloidosis Outcomes Survey (THAOS) registry of patients with ATTRv Glu89Gln amyloidosis, originating from Bulgaria and Italy, there were notable phenotypic differences in terms of organ involvement, with the cardiac phenotype more common in Bulgaria and the neurologic phenotype more common in Italy. Such differences could be explained by diverse factors: the symptom duration, first diagnosing physician, and other genetic and epigenetic factors (28). More than 30% of the patients with Glu89Gln had a mixed phenotype, suggesting a potential role of multiple genetic and/or environmental factors as well (28).

Although Val30Met is the most frequent *TTR* mutation and distributed worldwide, in Bulgaria it is the second most common. All the affected patients had a late-onset and mixed phenotype, similar to previous reports (29, 30).

Ser77Phe has the highest incidence in the French population (the south-western—Toulouse area). Historically, it can be speculated that this mutation has been transmitted during the Middle Ages by French crusaders, but still this has to be proven by a haplotype analysis. There is another hypothesis that it was transferred to France by the Bogomils after their expulsion from Bulgaria in the Middle Ages (31).

Gly47Glu was the most severe mutation in terms of age at the onset and rate of progression among the Bulgarian groups. In this respect, it is debatable what treatment to initiate even in the initial stages with very mild features (32, 33).

The majority (95%) of our patients showed a mixed phenotype (cardiac and neurological) at the time of diagnosis, regardless of their mutation.

Small unmyelinated fibers were affected earlier than the myelinated ones, which was proven by abnormal SSR and Sudoscan testing in all the symptomatic cases (24). SSR in our cohort seemed more sensitive than Sudoscan in the detection of the transition from an asymptomatic to a symptomatic state. Interestingly, we observed an advanced heart involvement even in patients with mild polyneuropathy at the time of diagnosis and *vice versa*, thus proving the presence of high heterogeneity in the same pedigree.

Our findings demonstrated that patients with Glu89Gln mutation may have a prolonged period of asymptomatic amyloid infiltration in the heart and the symptoms are further concealed by the development of the neuropathy. Thus, an earlier identification of the cardiomyopathy is needed through a close follow-up of patients and the mutation carriers. Many of the *TTR* pathogenic variants have heterogeneous phenotypic manifestation among different populations or even among the same family (28, 34, 35). The differences in organ and tissue involvement in the context of the same *TTR* variant imply the influence of other genetic and epigenetic factors upon the clinical manifestation. There are various studies proposing the effect of non-coding cis- and trans-acting regulatory genetic factors on the TTR expression and tissue-specific amyloid formation, as well as the age at disease onset (35–39).

Ocular manifestations were rare in the Bulgarian cohort (40–43). Decreased tear secretion was found in the majority of the cases, while amyloid deposition in the vitreous body and the lens, as well as bilateral ptosis were quite rare. Scalloped iris, glaucoma, and retinal vascular changes were not found in any of our 69 patients with ATTRv.

From our group treated patients with the TTR stabilizer for a period of 7 years 56% (84/150) remained stable, while in other 22.6% the neurological progression necessitated the treatment modification to TTR gene silencer (7). Those who progressed more rapidly or died, even on treatment, were diagnosed around 4 years after the clinical onset and had advanced restricted cardiomyopathy and heart failure.

The results from our study revealed that cardiac involvement has a great impact on prognosis as heart failure, sudden cardiac death, and cardio-embolic stroke are the main causes of death in the majority of the patients. In a previous study, we found a weak positive correlation between the NYHA class and the severity of neurological involvement in a cohort of patients with Glu89Gln mutation, i.e., a nonparallel progression of the polyneuropathy and the cardiomyopathy (8). The efficacy of Tafamidis treatment depends to a great extent on the severity of organ involvement at the time of treatment initiation, but also on the rate of disease progression, which depends on the type of the mutation but also on not fully elucidated individual patients' characteristics.

The follow-up of asymptomatic carriers in our group was initiated 10 years prior to the mean age at onset, characteristic for each mutation in the *TTR* gene (22). In the last 3 years, more than half of these asymptomatic carriers transitioned to symptomatic (22), and subsequently therapy with TTR stabilizers was initiated at a very early stage.

## Conclusion

Bulgarian patients with ATTRv display a mixed phenotype with some clinical peculiarities for each mutation, which should be considered when following up asymptomatic carriers of a specific gene defect. The screening programs of the high-risk patient population from the affected family or from the endemic region represent a potent tool for early diagnostic and timely treatment. The follow-up program for asymptomatic carriers has enabled us to initiate treatment as early as possible and, hopefully, with a better outcome for patients.

## Data Availability Statement

The raw data supporting the conclusions of this article will be made available by the authors, without undue reservation.

## Ethics Statement

The studies involving human participants were reviewed and approved by Medical University Sofia Institutional Review Board. Written informed consent for participation was not required for this study in accordance with the national legislation and the institutional requirements. Written informed consent was obtained from the individual(s) for the publication of any potentially identifiable images or data included in this article.

## Author Contributions

All authors contributed to manuscript preparation and critical review. All authors contributed to the article and approved the submitted version.

## Conflict of Interest

The authors declare that the research was conducted in the absence of any commercial or financial relationships that could be construed as a potential conflict of interest.

## Publisher's Note

All claims expressed in this article are solely those of the authors and do not necessarily represent those of their affiliated organizations, or those of the publisher, the editors and the reviewers. Any product that may be evaluated in this article, or claim that may be made by its manufacturer, is not guaranteed or endorsed by the publisher.
