# Supplementary material for: Seven Years of Selective Genetic Screening Program and Follow-Up of Asymptomatic Carriers With Hereditary Transthyretin Amyloidosis in Bulgaria
Source: Front Neurol. 2022 Apr 8;13:844595. doi: 10.3389/fneur.2022.844595 (PMC9024406; doi:10.3389/fneur.2022.844595)
Supplement: Supplementary file 1 [file Table_1.DOCX]

| ***TTR* mutation** | **Average carrier frequency in the screened family members** |
| --- | --- |
| c.325G>C, p.Glu109Gln (Glu89Gln) | 0.43 |
| c.290C>T, p.Ser97Phe (Ser77Phe) | 0.68 |
| c.148G>A, p.Val50Met, (Val30Met) | 0.65 |
| c.200G>A, p.Gly67Glu (Gly47Glu) | 0.23 |
| c.214T>C, p.Ser72Pro (Ser52Pro) | 0.50 |
| All ATTRv Positive families | 0.42 |

Table 1. Calculated average carrier frequency of the *TTR* mutations in the screened Bulgarian families with ATTRv.
